# Supplementary material for: Accuracy of 3D printed scan bodies for dental implants using two additive manufacturing systems: An in vitro study
Source: PLoS One. 2023 Apr 7;18(4):e0283305. doi: 10.1371/journal.pone.0283305 (PMC10081781; doi:10.1371/journal.pone.0283305)
Supplement: Attachment — Submitted filename: Responsestoreview_plos1_R2.docx [file pone.0283305.s002.docx]

RESPONSES TO REVIEW R2

COMMENT:

Introduction (2nd paragraph, line 5):

"An accuracy of a single implant PEEK scanbody is shown on average ~105-127 µm with 0.22°-1.25° in an *in vitro* study.[[13]](https://paperpile.com/c/IJIxCV/v1Kx9) An average range of ~36-57 µm was shown in an in vitro study for a single implant digital impression using a scan body-healing abutment combination system.[[14]](https://paperpile.com/c/IJIxCV/63JaQ)"

Please state in full for clarity purpose. For example the 'accuracy of a digitized single implant position transfer to a 3D printed cast or digital implant cast using PEEK scanbody'

RESPONSE: We appreciate the comment.

TEXT CHANGE: The statements were revised and clarified.

"An accuracy of a digitized single implant position transfer to a 3D printed cast or digital implant cast using PEEK scanbody of a single implant PEEK scanbody is shown on average ~105-127 µm with 0.22°-1.25° for an *in vitro* study.[[13]](https://paperpile.com/c/IJIxCV/v1Kx9) Similarly, an average range of ~36-57 µm was shown in an in vitro study for a single implant digital impression using a scan body-healing abutment combination system.[[14]](https://paperpile.com/c/IJIxCV/63JaQ)"

COMMENT:

The surmise in this study is 3D printing scanbodies which are not commercially available in market and checking their accuracy of dimensions and features in relation to the original piece. So, the authors also need to explicitly mention in the discussion that in order for this idea to develop in the future they at least need one (1) original scan body (physical) for them to duplicate (scan and create STL file) and 3D print for further use. So, this is a limitation if there is no available scanbody in the market for 3D Printing, unless the STL file can be directly acquired from the manufacturer. Also, the scanbody will vary for implant level and abutment level or tissue level/bone level implants of the same manufacturer. this needs to be emphasized too.

RESPONSE: Thank you very much for this important insight. We added the following clarification in the Discussion in the limitations.

TEXT CHANGE: The limitation statement and future studies statement were amended.

“Finally, this study utilized the known dimension of the manufacturer’s scan body. In real situations when a scan body is not available such as in the older implant systems or implants from other countries, the reverse-engineering process can be challenging and difficult to do. Additionally, variations from implant level or abutment level impression may also further complicate the design and fabrication process of the 3D printed scan body. Considering these limitations, future studies should include improving the design and printing trueness and precision prior to human clinical trials. Reverse-engineering of old dental implant parts as well as scan bodies will be an important topic as part of the future research.”
